# Safety assessment of the ethanolic extract of *Gongronema latifolium* Benth. leaves: a 90-day oral toxicity study in Sprague Dawley rats

**DOI:** 10.1186/s12906-019-2573-x

**Published:** 2019-06-28

**Authors:** Bassel Al-Hindi, Nor Adlin Yusoff, Mariam Ahmad, Item Justin Atangwho, Mohd Zaini Asmawi, Majed Ahmed Al-Mansoub, Yasser Mahfooth Tabana, Idris Bello, Mun Fei Yam

**Affiliations:** 1 School of Pharmaceutical Sciences, Universiti Sains Malaysia, 11800 Penang, Malaysia; 2 Integrative Medicine Cluster, Advanced Medical and Dental Institute, Universiti Sains Malaysia, Bertam 13200 Kepala Batas, Penang, Malaysia; 3 Department of Biochemistry, College of Medical Sciences, University of Calabar, Calabar, Nigeria; 4 Faculty of Pharmacy and Pharmaceutical Sciences, University of Alberta, 116 St & 85 Ave, Edmonton, AB T6G 2R3 Canada

**Keywords:** *Gongronema latifolium* Benth, Ethanol extract, Plant medicine, Subchronic toxicity

## Abstract

**Background:**

The leaves of *Gongronema latifolium* Benth. have long been recognized traditionally as a remedy for a variety of ailments in Africa. This study was conducted to evaluate the safety profile of the ethanolic extract of *G. latifolium* (GLES) leaves through a repeated dose 90-day oral toxicity study in male and female of Sprague Dawley rats.

**Methods:**

GLES was orally administered at doses of 250, 500 and 1000 mg/kg/day consecutively for 90 days.

**Results:**

No behavioral or physiological changes and mortality were observed. GLES did not have a marked impact on general hematological parameters and did not precipitate nephrotoxicity. However, compared to the control, serum triglycerides, total cholesterol and low-density lipoprotein levels were lower and white adipose tissue paired retroperitoneal fat depots were depleted in male rats treated with GLES3 by the end of the experiment. The liver was significantly enlarged in GLES-treated rats of both sexes. Negative gender-specific alterations were observed with the highest dose. Adverse risk was evident in the female rats mainly due to marked body weight gain and cerebrum weight reduction.

**Conclusion:**

Further research is needed to reach more specific conclusions about to the safety of ingesting high doses of GLES for long periods of time.

## Background

*Gongronema latifolium* Benth. (Asclepiadaceae) (GL) is widespread in the tropical and subtropical regions of Africa. It is known to the natives as “Utazi” and “Arokeke” in south eastern and south western Nigeria, respectively [1, 2]. It is also found in South America, and has moderate representation in north and south east Asia [3]Gongronema latifolium has long been recognized as an African traditional remedy for a variety of ailments, such as hypertension, diabetes mellitus, malaria, mental and intestinal disorders [1, 4]. In the United States, GL leaves are incorporated into a tea blend that is mainly marketed to diabetes mellitus patients [5]. Several pharmacological activities of GL extracts have been studied and reported, which provided experimental support for the empirical ethnopharmacological use of this plant in folk medicine. For example, anti-inflammatory [6], antifungal [7], anti-laxative [8] and antidiabetic [9, 10] activities have been reported. Over the past two decades, different parts of GL have been found to contain saponins, anthraquinones, alkaloids, β-sistosterol, sitostenone, lupenyl esters, pregnance ester, glucosides, and essential oils [10–12].

Despite the extensive traditional uses of GL, related scientific reports, increasing research interest, and growing demand for GL, few detailed studies of the short-term safety and/or chronic toxicity of the use of GL have been conducted. Gamaniel and Akah who studied the acute toxicity of aqueous extract of the stem of GL in mice estimated the intraperitoneal median lethal dose (LD_50_) value to be 1678.63 mg/kg body weight (BW) [8]. Similar study was conducted by Sylvester et al. on the ethanol leaf extract of GL and oral LD_50_ value was reported to be 1500 mg/kg body BW [13]. Effiong et al. performed acute and subacute (30 and 60-day) toxicity studies of an ethanolic leaf extract of GL (GLES) in rodents. They reported that the acute oral LD_50_ of GL exceeded 5 g/kg BW, and GL was not toxic at doses below 300 mg/kg [14].

The present study was designed following relevant Organisation for Economic Co-Operation and Development (OECD) guidelines to assess the effects of 90-day repeated oral administration of a range of doses of GLES in female and male Sprague Dawley (SD) rats. Rats are used in this study because they are small mammals that are easy to handle, calm and their physiology are more like the corresponding human condition [15]. In addition to that, SD rat has been widely used as a model to study the toxicity effect of medicinal plants [16, 17]. Adverse findings were highlighted, and No Observed Adverse Effect Level (NOAEL) values were estimated for each gender separately. Results can be by researchers and health care professionals to assess possible health risks of long-term exposure to high doses of GLES.

## Methods

### Plant material

GL was collected as a whole plant from Yakkur, Cross River State, Nigeria (6° 08′17.35″N 8° 41′15.54″E, elevation 420 ft). The plant was authenticated by Pastor Frank, a botanist in the Department of Botany, University of Calabar and a voucher specimen (ERU/2011/718) was deposited at the same department. The leaves were plucked from the plant, washed with tap water and dried in the shade. Similar drying practice was conducted by the locals. The dried leaves were ground into powder, properly packaged and sent by courier to the Department of Pharmacology, Universiti Sains Malaysia (USM), Penang, Malaysia. The powder was received within 7 days.

### Preparation of plant extract

Upon receipt of the sample, 400 g of powdered dried GL were extracted using a Soxhlet apparatus at 40–60 °C at a ratio of 1:5 to 1:10 of material:ethanol (w/v) for 3 days. The extract was concentrated to about one tenth of its original volume in a rotary evaporator (Buchi Labortechnik, AG CH-9230 Flawil, Switzerland) at 40 °C. Thereafter, the concentrate was freeze-dried to obtain the dried extract (yield: 9.45%). The dried sample was stored at 4 °C until further use.

### Animals and housing

Eighty SD rats (40 males and 40 females) were obtained from the Animal Research and Service Centre, USM. The animals were acclimatized for 5 days prior to the experiment in the Animal Transit Room, School of Pharmaceutical Sciences, USM. The animals were 6–7 weeks old with the body weight ranged from 180 to 220 g upon commencement of the experiment. The rats were housed pair-wise under standard environmental conditions (temperature, 25 ± 5 °C; relative humidity, 50 ± 5% and a 12 h light/dark cycles) in the ventilated polycarbonate cages (Tecniplast, 480 × 375 × 210 mm) throughout the period of the experiment. The animals were allowed free access to standard rat pellets (Gold Coin Feedmills, Butterworth, Penang, Malaysia) and tap water ad libitum. Care and handling of study animals were performed according to the guidelines set by the World Health Organization (WHO, Geneva, Switzerland) with consideration of the principles of the Hungarian Act 2011 CLVIII (modification of Hungarian Act 1998 XXVIII) regulating animal protection.

### 90-day oral toxicity study

The study was carried out according to OECD test guideline 408 (90-day study) and US Food and Drug Administration (FDA) Redbook 2000, IV.C.4.a (90-day study) [18]. On the last day of acclimatization, rats were divided randomly according to the body weight, such that mean body weight difference of rats did not exceed ±20% of the mean body weight of each sex group. A total of 80 SD rats (40 males and 40 females) were divided into four groups, one control group and three treatment groups, with each group containing 20 rats (10 males and 10 females). GLES was dissolved in 4% Tween-80 (Sigma-Aldrich, USA) and administered orally daily for 90 days at single doses of 250 (GLES1), 500 (GLES2) and 1000 (GLES3) mg/kg BW, while the control group received the vehicle only (Table 1). The dosing time at approximately 11 a.m. for the rats were maintained over the study period to minimize.

**Table 1:** Animal grouping for treatment with ethanolic extract of *G. latifolium*

Group	No. of animals (*n*)	Treatment
GLES1	10 males, 10 females	250 mg/kg BW of GLES daily
GLES2	10 males, 10 females	500 mg/kg BW of GLES daily
GLES3	10 males, 10 females	1000 mg/kg BW of GLES daily
Control	10 males, 10 females	10 ml/kg BW of the vehicle daily

the biological variation among the rats.

### Observational study

During the study period, the clinical and behavioral signs of toxicity and event of mortality were closely monitored twice daily. The following clinical signs were assessed: changes in eyes, skin, fur, mucous membranes, secretions and excretions. Also, behavioral examination included writhing, repetitive circling, bizarre behavior, posture and response to handling were noticed. The body weight of the animals and food consumption were recorded twice a week.

### Hematology and biochemistry analyses

At the end of the 90-day period, the animals were fasted overnight and inhalational anesthesia was conducted using 2% isoflurane (Merck KGaA, Darmstadt, Germany) in an induction chamber to allow blood to be collected via cardiac puncture. A portion of the collected blood was dispensed into tubes containing ethylene diamine tetra acetic acid (EDTA) and the remainder was place in plain tubes for hematology and biochemistry analyses, respectively. The analyses were performed at Gribbles Pathology (M) Sdn. Bhd., Penang, Malaysia. The following hematological parameters were assessed using a Sysmex KX-21 N Hematology Analyzer (Sysmex Corporation, Kobe, Japan): white blood cells (WBC), red blood cells (RBC), hemoglobin (HGB), platelets (PLT), packed cell volume (PCV), mean corpuscular volume (MCV), mean corpuscular hemoglobin (MCH), mean corpuscular hemoglobin concentration (MCHC), lymphocyte absolute value (LYM), neutrophils absolute value (NEU), and red cell distribution width (RDW). Serum biochemical analyses of level of alanine aminotransferase (ALT), aspartate aminotransferase (AST), alkaline phosphatase (ALP), total protein (TP), albumin (ALB), globulin (GLO), blood urea nitrogen (BUN), creatinine (CREA), calcium (Ca), and phosphorus (P); were performed using an automated chemistry analyzer (Olympus AU640 Chemistry Immuno-Analyzer, Tokyo, Japan). Lipid profiles namely total cholesterol (CHOL), high density lipoprotein cholesterol (HDLC), low density lipoprotein cholesterol (LDLC) and triglycerides (TG) were determined using an ADVIA 2400 Chemistry Analyzer (Siemens, Erlangen, Germany) and very low density lipoprotein cholesterol (VLDLC) concentrations were calculated as follows using Friedewald’s equation [19]:

VLDLC (mM/L) = Triglyceride / 5.

The atherogenic indices were calculated as follow:

Cardiac Risk Ratio (CRR) = CHOL/HDLC [20].

Castelli’s Risk Index-2 (CRI-2) = LDLC/HDLC [21].

Atherogenic Coefficient (AC) = (CHOL – HDLC)/HDLC [22].

Atherogenic Index of Plasma (AIP) = log (TG/HDLC) [23].

### Histopathology study

After cardiac puncture, animals were immediately sacrificed by cervical dislocation. Necropsy was performed carefully, and the following tissues/organs were isolated and weighed: liver, kidneys, adrenal glands, spleen, adipose tissue (paired retroperitoneal pads), heart, lungs, cerebrum, thymus, stomach, gut, uterus, and ovaries (or testes). Paired organs were weighed together. The relative organ weights were calculated based on the organ to body weight ratio. Vital organs (liver and kidneys) were fixed for histological study. Isolated kidneys and livers from the 10 males and 10 females per group were fixed in 10% buffered formalin, embedded in paraffin and sectioned into 4- to 6-μm sections before being stained with hematoxylin-eosin [24]. The tissues were visualized using a Leica MZ6 optical microscope (Leica Microskopie und Systeme, Germany) equipped with a Leica Qwin (Leica Imaging Systems, Cambridge, England).

### Statistical analysis

Data obtained from the male and female treatment groups were compared separately. The statistical comparison was aimed at determining whether the differences observed between the treatment groups and the control resulted from GLES consumption. Results were expressed as the mean ± standard error of the mean (SEM). Statistical analysis was performed using version 21 of the IBM-SPSS statistical program (IBM Corp., Armonk, NY, USA). One-way analysis of variance was used followed by Dunnett’s test for parametric multiple comparisons between the control and treatment groups. Differences were considered significant at *p* < 0.05.

## Results

### Effect of 90-day oral administration of GLES on general behavior of rats

**Fig. 1:**
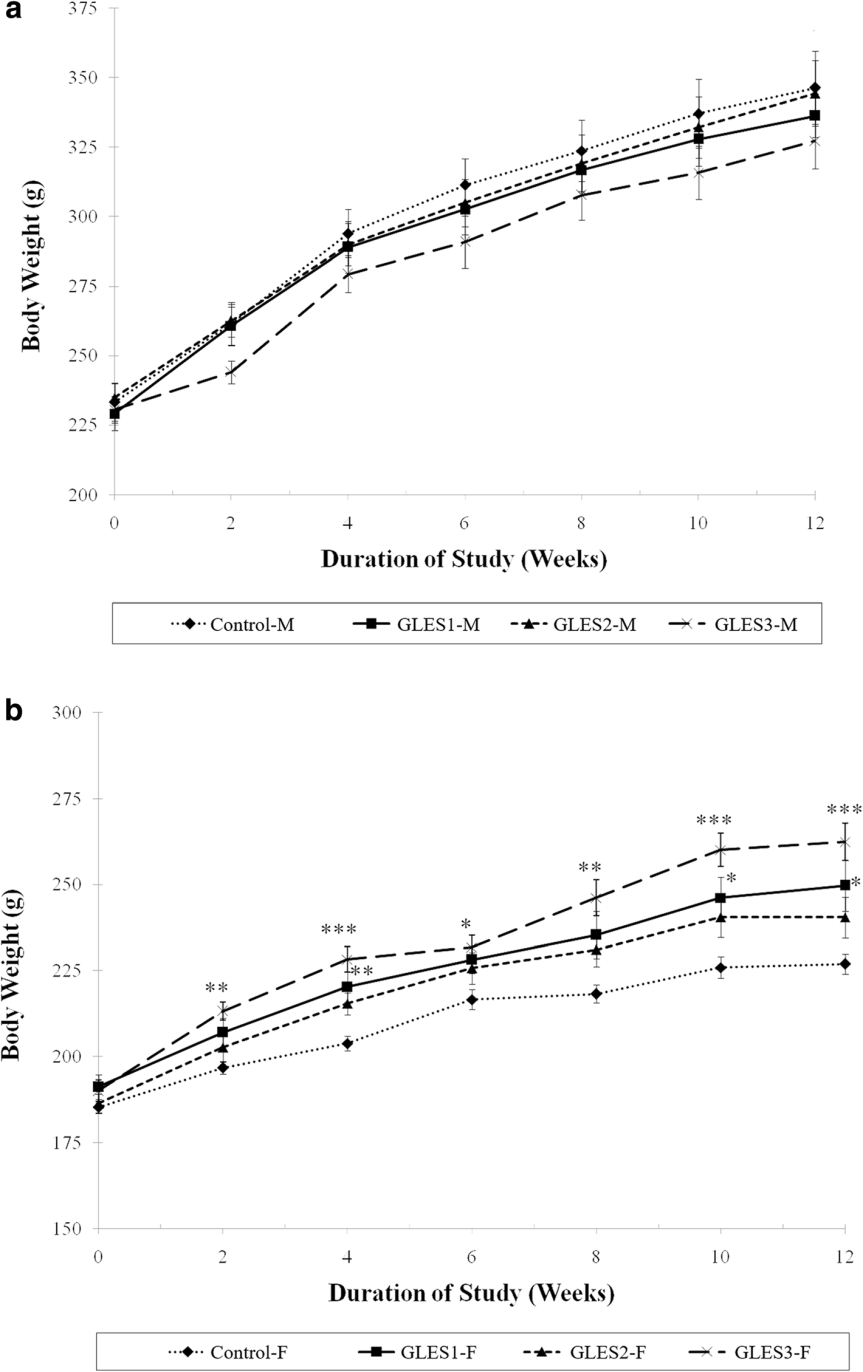
Mean weight of rats treated orally with GLES for 90 days: (**a**) male rats (**b**) female rats; *, **, and *** indicate significant differences (*p* < 0.05, *p* < 0.01 and *p* < 0.001, respectively) in weight gain compared with the control

There were no deaths recorded during or at the end of the 90-day treatment with GLES. The animals were observed twice daily and no adverse effects on animal behavior or physical appearance were observed throughout the study. Body weight and food consumption were measured twice weekly. The growth patterns in the three male treatment groups were not different from that of the control (Fig. 1). However, the rate of body weight gain of female GLES3 rats was significantly higher than the rate of the control throughout the study period, although there were no significant changes in food consumption compared with the control. Rats in the female GLES1 group showed significantly grater weight gain than the control at 4, 10, and 12 weeks.

### Effect of 90-day oral administration of GLES on relative organ weights of rats

Relative organ weights were measured upon termination of the study in both rat genders (Table 2). Compared to the control, the livers of the females were significantly enlarged in a dose-dependent manner, as was also true for the adrenals of GLES3 group (*p* < 0.05). Male rats in groups GLES2 and GLES3 had enlarged livers (*p* < 0.01). In contrast, the paired retroperitoneal body fat depots of the male rats were significantly depleted in a dose-dependent manner compared to the control (*p* < 0.01). The weight of the cerebrum in female rats groups GLES1 and GLES3 was significantly lower than that of the control (*p* < 0.05). Moreover, the testes weight of male rats in the GLES3 group were significantly lower than that of the control (*p* < 0.05).

**Table 2:** Relative organ weights of rats treated orally with GLES for 90 days

	Control		GLES1		GLES2		GLES3	
	Female	Male	Female	Male	Female	Male	Female	Male
Relative weight (g/100 g Body weight)								
Liver	2.91 ± 0.07	2.42 ± 0.07	3.34 ± 0.08*	2.81 ± 0.15	3.49 ± 0.15**	3.14 ± 0.15***	3.72 ± 0.13***	3.09 ± 0.12**
Kidneys	0.57 ± 0.01	0.59 ± 0.02	0.57 ± 0.02	0.60 ± 0.02	0.57 ± 0.01	0.64 ± 0.02	0.56 ± 0.012	0.57 ± 0.01
Spleen	0.19 ± 0.01	0.19 ± 0.01	0.18 ± 0.01	0.17 ± 0.01	0.19 ± 0.01	0.19 ± 0.01	0.19 ± 0.01	0.16 ± 0.01
Adipose tissue	0.77 ± 0.07	0.72 ± 0.07	0.85 ± 0.12	0.46 ± 0.06**	0.63 ± 0.07	0.42 ± 0.05**	0.57 ± 0.04	0.38 ± 0.03***
Heart	0.33 ± 0.01	0.30 ± 0.006	0.33 ± 0.01	0.30 ± 0.007	0.33 ± 0.01	0.30 ± 0.004	0.30 ± 0.01	0.28 ± 0.007
Lungs	0.78 ± 0.06	0.56 ± 0.03	0.83 ± 0.13	0.56 ± 0.02	0.69 ± 0.02	0.53 ± 0.02	0.69 ± 0.04	0.55 ± 0.04
Cerebrum	0.60 ± 0.01	0.40 ± 0.02	0.51 ± 0.02*	0.39 ± 0.01	0.56 ± 0.03	0.38 ± 0.02	0.45 ± 0.02***	0.40 ± 0.01
Thymus	0.10 ± 0.01	0.08 ± 0.01	0.12 ± 0.01	0.07 ± 0.01	0.09 ± 0.01	0.07 ± 0.01	0.11 ± 0.01	0.07 ± 0.01
Adrenal glands	0.019 ± 0.001	0.016 ± 0.002	0.021 ± 0.002	0.015 ± 0.002	0.021 ± 0.001	0.015 ± 0.001	0.023 ± 0.001*	0.015 ± 0.001
Ovaries (or testis)	0.037 ± 0.002	0.94 ± 0.01	0.041 ± 0.001	0.89 ± 0.03	0.037 ± 0.002	0.87 ± 0.04	0.041 ± 0.001	0.83 ± 0.04*
Uterus	0.29 ± 0.02	N/A	0.24 ± 0.02	N/A	0.24 ± 0.01	N/A	0.26 ± 0.02	N/A

*n* = 10 rats/sex/group; data are presented as mean ± SEM

*indicates significant difference (*p* < 0.05) compared to the control

**indicates significant difference (*p* < 0.01) compared to the control

***indicates significant difference (*p* < 0.001) compared to the control

### Effect of 90-day oral administration of GLES on hematological and biochemical indices in rats

Hematological parameters, serum liver and kidney parameters, and the lipid profile of the male and female rats were measured upon termination of the study. For the hematological assessment, no significant effects on the measured blood indices were detected in the treatment groups compared to the control, except for an increase in RBC count (*p* < 0.01) in the female rats treated with the highest dose of the extract (GLES3) (Table 3).

**Table 3:** Hematology values in rats treated orally with GLES for 90 days

	Control		GLES1		GLES2		GLES3	
	Female	Male	Female	Male	Female	Male	Female	Male
WBC (*10^9^/L)	6.08 ± 0.81	9.47 ± 1.62	6.96 ± 0.85	8.87 ± 1.26	5.37 ± 0.73	8.87 ± 1.09	8.09 ± 1.15	7.47 ± 0.86
RBC (*10^12^/L)	7.20 ± 0.24	8.92 ± 0.13	7.64 ± 0.12	8.92 ± 0.25	7.29 ± 0.15	8.67 ± 0.13	7.99 ± 0.15**	8.45 ± 0.23
HGB (g/L)	137.30 ± 1.64	152.20 ± 1.56	142.50 ± 1.97	151.90 ± 2.70	134.20 ± 3.33	146.60 ± 2.03	144.10 ± 2.61	147.70 ± 1.92
PLT (*10^9^/L)	865.70 ± 36.62	875.80 ± 48.90	824.10 ± 37.34	901.60 ± 43.82	918.50 ± 79.82	953.50 ± 61.47	968.5 ± 43.83	931.20 ± 71.60
PCV (*10^12^/L)	0.42 ± 0.01	0.45 ± 0.01	0.45 ± 0.01	0.46 ± 0.02	0.43 ± 0.01	0.43 ± 0.01	0.44 ± 0.01	0.43 ± 0.01
MCV (fL)	58.60 ± 1.00	50.60 ± 1.18	58.40 ± 0.96	51.20 ± 1.81	58.60 ± 1.23	49.70 ± 1.09	55.50 ± 0.64	51.40 ± 1.30
MCH (pg)	19.40 ± 0.86	17.20 ± 0.20	18.50 ± 0.22	17.20 ± 0.29	18.30 ± 0.26	16.90 ± 0.23	18.10 ± 0.23	17.50 ± 0.34
MCHC (g/L)	329.50 ± 11.63	338.90 ± 5.98	319.70 ± 1.71	336.10 ± 8.46	315.00 ± 3.96	341.40 ± 5.31	325.30 ± 2.19	342.10 ± 5.39
LYM# (*10^3^/μL)	4.09 ± 0.63	6.12 ± 0.93	3.62 ± 0.85	6.41 ± 0.92	3.68 ± 0.53	5.70 ± 0.81	5.45 ± 0.75	4.93 ± 0.59
NEU# (*10^3^/μL)	1.47 ± 0.20	2.64 ± 0.66	1.78 ± 0.26	1.92 ± 0.37	1.23 ± 0.20	2.35 ± 0.41	1.80 ± 0.31	2.08 ± 0.27
RDW (%)	14.32 ± 0.31	18.01 ± 0.23	14.35 ± 0.56	18.42 ± 0.38	15.02 ± 0.66	17.90 ± 0.36	14.37 ± 0.22	16.82 ± 0.59

*n* = 10 rats/sex/group; data are presented as mean ± SEM

**indicates significant difference (*p* < 0.01) between GLES3 and the control

Compared to the control, GLES exerted no statistically significant effect on the kidney function or the liver parameters measured (Table 4).

**Table 4:** Serum liver and kidney parameters in rats treated orally with GLES for 90 days

	Control		GLES1		GLES2		GLES3	
	Female	Male	Female	Male	Female	Male	Female	Male
ALT (IU/L)	50.80 ± 3.97	60.70 ± 4.38	51.60 ± 1.29	62.50 ± 3.43	49.80 ± 3.54	60.80 ± 2.97	48.60 ± 2.59	58.50 ± 2.81
AST (IU/L)	157.00 ± 19.56	216.10 ± 21.63	127.60 ± 8.39	208.00 ± 17.10	172.10 ± 19.46	210.20 ± 21.85	113.30 ± 10.88	170.40 ± 16.58
TP (g/L)	81.40 ± 1.64	70.90 ± 1.73	82.30 ± 1.48	73.90 ± 1.55	85.00 ± 1.82	72.20 ± 1.58	81.10 ± 0.90	73.70 ± 1.22
ALB (g/L)	35.10 ± 0.89	32.40 ± 1.28	34.10 ± 0.66	32.00 ± 1.51	36.70 ± 0.63	33.40 ± 1.28	34.60 ± 0.58	33.60 ± 1.24
ALP (IU/L)	115.00 ± 14.42	123.70 ± 10.51	143.50 ± 18.45	128.30 ± 16.34	88.90 ± 14.48	138.50 ± 20.32	109.30 ± 10.46	108.50 ± 7.30
GLO (g/L)	46.30 ± 1.14	38.50 ± 2.46	48.20 ± 1.93	41.90 ± 2.11	48.30 ± 1.63	38.80 ± 2.46	46.50 ± 0.54	40.10 ± 2.33
BUN (mM/L)	5.56 ± 0.30	7.32 ± 0.19	5.73 ± 0.17	7.76 ± 0.25	5.85 ± 0.41	7.75 ± 0.24	4.88 ± 0.19	7.92 ± 0.25
CREA (mM/L)	39.60 ± 2.16	35.60 ± 3.59	39.10 ± 1.17	35.90 ± 3.99	42.00 ± 2.65	32.10 ± 3.21	39.60 ± 0.88	32.10 ± 1.87
Ca (mM/L)	2.78 ± 0.03	2.44 ± 0.03	2.79 ± 0.03	2.47 ± 0.02	2.73 ± 0.02	2.47 ± 0.02	2.72 ± 0.03	2.51 ± 0.02
P (mM/L)	2.25 ± 0.09	2.81 ± 0.05	2.40 ± 0.09	2.79 ± 0.11	2.17 ± 0.08	2.75 ± 0.11	2.07 ± 0.09	2.70 ± 0.06

*n =* 10 rats/sex/group; data are presented as mean ± SEM

However, the extract affected some of the measured lipid indices significantly, especially in the male rats (Table 5). Compared with the control, serum CHOL, TG and VLDLC levels were significantly (*p* < 0.001) lower in the male animals that received 500 and 1000 mg/kg BW of GLES. Furthermore, LDLC levels were significantly (*p* < 0.05) lower (by about 40%) in the male animals treated with 1000 mg/kg BW of GLES. In the female rats, significant (*p* < 0.05) increases of HDL and CHOL levels were observed in GLES3 compared to the control. However, translating these changes into clinical risk indicators showed that GLES administered up to 1000 mg/kg BW had no significant impact on the CRR, CRI-2, AC or AIP.

**Table 5:** Serum lipid profile in rats treated orally with GLES for 90 days

	Control	GLES1		GLES2		GLES3		
	Female	Male	Female	Male	Female	Male	Female	Male
CHOL (mg/dL)	72.97 ± 5.41	63.71 ± 3.47	69.88 ± 3.47	55.21 ± 3.09	84.17 ± 6.18	44.02 ± 1.54***	91.51 ± 4.63*	44.40 ± 2.70***
TG (mg/dL)	30.12 ± 3.47	19.69 ± 1.93	38.61 ± 6.18	14.67 ± 1.16*	28.57 ± 3.09	11.97 ± 1.16***	28.96 ± 3.09	9.65 ± 0.77***
HDL-C (mg/dL)	52.90 ± 3.47	20.85 ± 4.25	49.42 ± 2.32	22.39 ± 3.86	61.78 ± 4.63	14.29 ± 3.47	67.95 ± 3.47*	19.31 ± 4.25
LDL-C (mg/dL)	6.95 ± 2.32	33.98 ± 3.47	4.25 ± 1.54	26.25 ± 2.70	9.65 ± 2.70	24.32 ± 3.09	10.42 ± 1.54	20.46 ± 2.70**
VLDLC (mg/dL)	6.02 ± 0.69	3.94 ± 0.35	7.68 ± 1.27	2.90 ± 0.23*	5.75 ± 0.66	2.39 ± 0.19***	5.79 ± 0.58	1.89 ± 0.12***
CRR	1.37 ± 0.018	4.45 ± 0.80	1.41 ± 0.02	3.45 ± 0.67	1.37 ± 0.02	4.88 ± 0.88	1.35 ± 0.02	3.53 ± 0.69
CRI-2	0.12 ± 0.03	2.81 ± 0.69	0.09 ± 0.03	2.04 ± 0.59	0.14 ± 0.04	3.30 ± 0.77	0.15 ± 0.02	2.18 ± 0.62
AC	0.37 ± 0.02	3.44 ± 0.80	0.41 ± 0.02	2.45 ± 0.67	0.37 ± 0.02	3.88 ± 0.88	0.35 ± 0.02	2.53 ± 0.69
AIP	−0.263 ± 0.061	0.058 ± 0.089	−0.147 ± 0.051	−0.115 ± 0.083	−0.346 ± 0.073	0.019 ± 0.010	−0.382 ± 0.051	−0.210 ± 0.097

*n =* 10 rats/sex/group; data are presented as mean ± SEM

*indicates significant difference (*p* < 0.05) compared to the control

**indicates significant difference (*p* < 0.01) compared to the control

***indicates significant difference (*p* < 0.001) compared to the control

### Gross necropsy and histopathology examination of the liver and kidneys

**Fig. 2:**
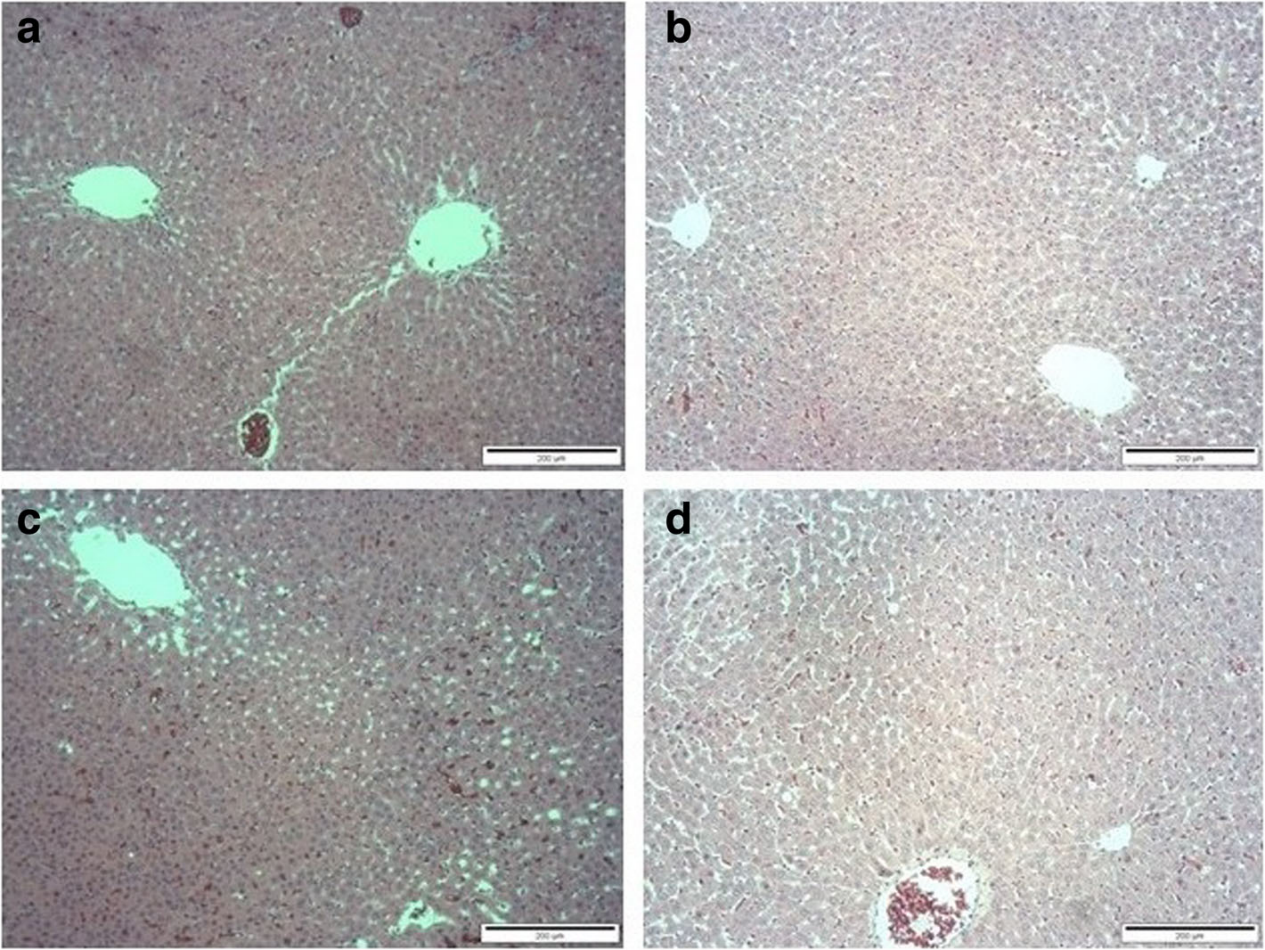
Effect of 90-day oral administration of GLES on liver histomorphology in rats: (**a**) Control group of male rats; (**b**) Control group of female rats; (**c**) GLES3-treated group of male rats; (**d**) GLES3-treated group of female rats. Original magnification 100x

**Fig. 3:**
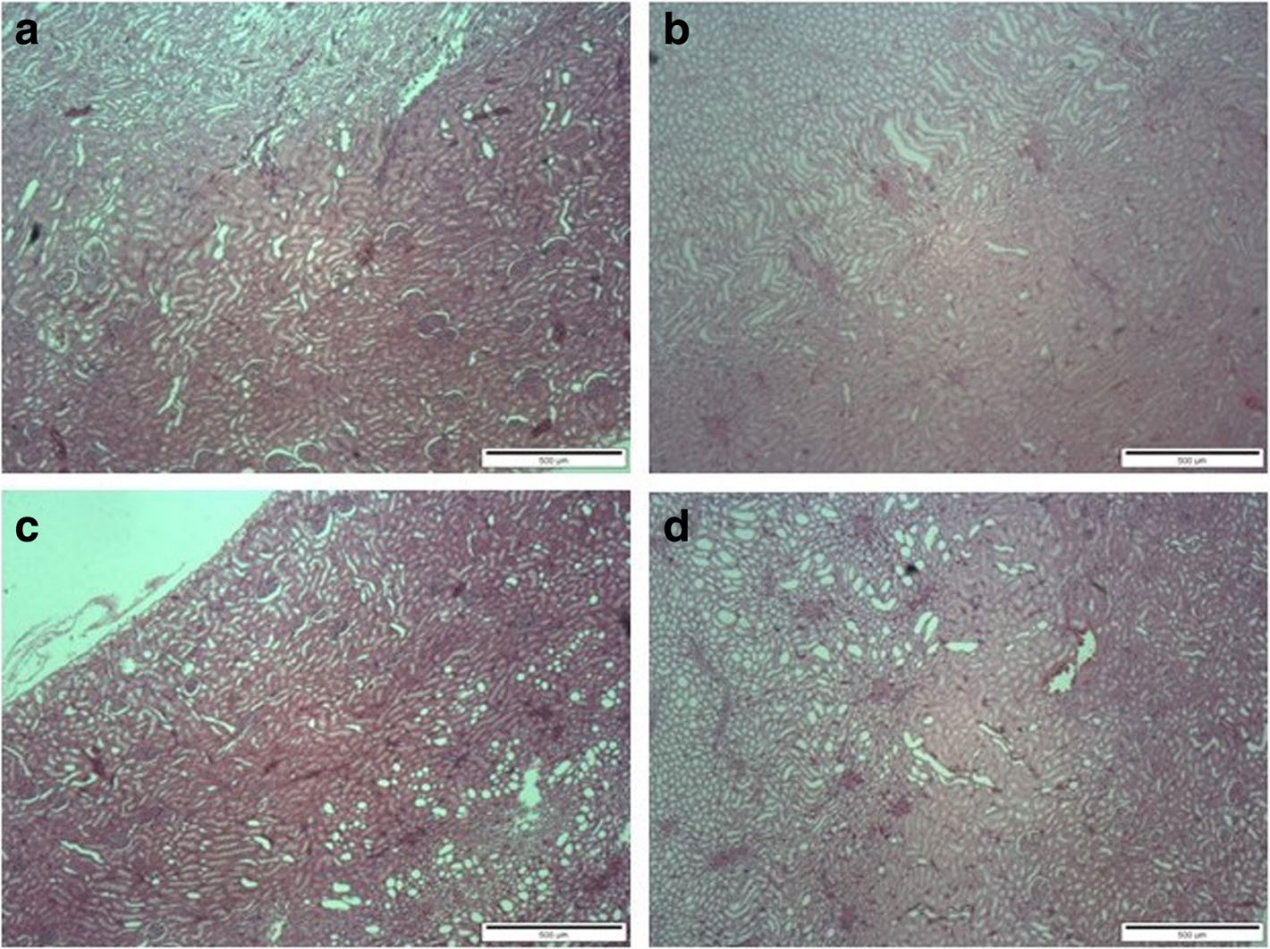
Effect of 90-day oral administration of GLES on kidney histomorphology in rats: (**a**) Control group of male rats; (**b**) Control group of female rats; (**c**) GLES3-treated group of male rats; (**d**) GLES3-treated group of female rats. Original magnification 40×

There were no gross pathological lesions found during necropsy. However, treatment-related histopathological alterations were found in the liver of the male SD rats, especially those receiving GLES at the highest dose (1000 mg/kg BW/day) for 90 consecutive days. Findings included hemorrhage and necrotic vacuoles (Fig. 2). The histology of the kidneys appeared normal in both genders (Fig. 3).

## Discussion

The WHO estimates that 70–80% of the people in developing countries use traditional medicine as a major source of health care [25]. GL is a herb that has long been an integral part of African traditional medicine and its beneficial properties have been well documented, especially as an antidiabetic herb [1, 9, 11, 26, 27]. In this study, we investigated an ethanolic extract of GL because similar extracts have been reported to be the most efficacious in exerting the anti-diabetic effect [28].

The rat is the species of choice in most preclinical toxicological studies that aim to evaluate pharmaceutical candidates [29]. Hence, SD rats (10 per sex per group) were selected and treated with GLES during a 90-day period in compliance with OECD and FDA guidelines for repeated dose oral toxicity studies. The 90-day repeated oral toxicity study is essential for assessment of the safety of GL particularly when it is incorporated into the daily diet. Throughout the study period, no death or remarkable changes in the normal physical activity or behavior of the studied animals. Similarly, Effiong et al. and Sylvester et al. reported no mortality in acute oral toxicity tests of ethanol extract of GL leaf with doses up to 8000 mg/kg BW [13, 14].

Oral consumption of up to 1000 mg/kg BW of GLES for 90 days did not cause alterations in the weight of the spleen, heart, lungs or thymus glands in rats of either sexes, while liver enlargement was seen in both sexes. However, gender-specific adverse risk was evident in the male rats in the form of decreased retroperitoneal fat and testes weight, and in the female rats in the form of increased weight of the adrenal glands and decreased cerebrum weight compared to normal control rats. A significant changes in organ weight is a well-known indicator of chemically induced changes to organs [30]. Alterations in normal body weight suggest impairment of some bodily or organ functions. For example, change in liver weight may indicate hepatocellular hypertrophy [31], elevated adrenal gland weight may suggest hyperplasia, hypertrophy or atrophy [32] while variation in testes weights may associate with changes in seminiferous or interstitial edema [33]. Such a weight impact could be worse in diabetic animals given GLES on a long-term basis. Thus, further evaluation of the effect of GLES on male and female BW in type 2 diabetic conditions is needed.

The observed decrease in cerebrum weight (absolute and relative) confirms that GLES affected brain weight in the female rats and rules out the possibility that the observed decreased was only due to coincidental body weight abnormalities. Ekong et al. [34] reported that combination of ethanol extracts of GL leaf and *Rauwolfia vomitoria* root ameliorated cerebral degeneration in a 7-day study, and Ekong et al. [4] showed that an ethanol extract of GL leaf given alone caused cerebral cytoarchitectural changes with no effect on the weight of the brain after 7 days of treatment. Again, when ethanol extract of GL leaf was administered in combination with ethanol extract of *Rauwolfia vomitoria* root, an increase in the cerebellar cortical cellular was observed [35]. Perhaps, results of the present study may encourage researchers to investigate the effects of GL on cerebral architecture and weight more selectively and comprehensively.

The highest GLES dose tested in this study resulted in the greatest adverse effect. The liver was enlarged in both sexes compared with the control rats. According to Adenuga et al., liver hypertrophy may be adaptive in nature, or idiopathic [36]. However, treatment-related increases in the weight of the liver can imply a wide variety of causes, such as hyperplasia of a resident cell type, hypertrophy, inflammation, fibrosis, abnormal storage of metabolism products, particles, or cleavage products, neoplasia and/or congestion [37, 38]. Although the increase observed in the current study was below the 150% liver hypertrophy that Lewis et al. recommended as a limit (indicative of increased hepatocarcinogenic risk) [39], it was treatment-related, at least for the male rats, as liver histology revealed the presence of necrotic vacuoles and hemorrhage [40]. However, no enzymatic alterations in the liver-related parameters were detected, which may suggest that injury was not extensive enough to cause them. Nevertheless, it is well known that the lack of enzymatic abnormality does not necessarily confirm the absence of hepatic disease [41].

Results of kidney function tests, especially the level of creatinine, are key indicators of potential toxicity. An elevated creatinine level may indicate impaired glomerular filtration and kidney damage [42]. In this study, nephrotoxicity was absent as indicated by normal relative kidney weight, histology and serum creatine levels.

Alterations in hematological parameters are particularly useful to assess toxic effects in animal studies [43]. In this study, GLES did not have a marked impact on general haematological parameters of the male rats compared to the control group, suggesting that GLES has no adverse effect on the hematopoiesis process. In the female rats treated with the highest dose of GLES, however, a significant (*p* < 0.01) increase in the RBC count was observed. Further research is needed to confirm whether long-term administration of a high dose of GLES has any effect on the bone marrow.

Changes in the normal metabolism of studied animals can be evaluated using the lipid profile [44]. In this study, GLES treatment resulted in an increase in HDL with no abdominal fat decrease in the female rats. Serum TG, CHOL, and LDLC levels were lower and white adipose tissue (WAT) paired retroperitoneal fat depots were significantly (*p* < 0.05) depleted in GLES-treated male rats versus control male rats. Absolute adipose tissue weight was statistically different among the male groups, which confirmed a connection between the reduction in adipose tissue weight and the lipid indices cited above. WAT is the main storage site for energy in the form of lipids in the body [45]. It is a prime factor in obesity and contributes to insulin resistance by decreasing insulin-stimulated glucose disposal in the skeletal muscles [46], which are the main consumer of glucose in vivo [47]. Unlike subcutaneous fat, surgical removal of visceral pads has been shown to improve metabolic parameters, and the mean and maximum lifespan of rats [48]. To our knowledge the visceral fat-depleting property of GLES is novel and can be exploited to deepen our understanding of the antihyperglycemic and antihyperlipidemic effects of GL.

The gender-specific findings detected in this study may indicate possible hormonal interactions with the steroidal components of GLES. Steroidal components have been found in GL extracts [10–12], and GL is traditionally used to restore a normal menstrual cycle in women post-partum [8]. The NOAEL values estimated separately for each gender were 250 mg/kg BW for male SD rats and < 250 mg/kg BW for female SD rats.

There are always variations that make the comparison of results between studies tricky. The findings presented herein are contradictory to the small number of previous toxicity reports for GL. Those studies tested low doses over shorter treatment periods and did not report any major adverse effects. Studies conducted in the geographical location where a medicinal plant is native are often over-optimistic and prone to publication bias [49]. Moreover, researchers tend to adopt a safe approach when investigating the toxicity of vegetables/spices traditionally incorporated in the daily diet. That being said, extracts tend to vary in properties and toxic potential depending upon many parameters, such as geographical source of the materials, time of harvest, soil conditions [50], extraction procedure used, the solvent [51], and the presence of contaminants. Additionally, some herbal medicines have been shown to have severe adverse effects despite being generally labeled as safe due to poor regulatory policies and the lack of long-term clinical data [52].

The present study was not a comprehensive investigation and suffers from several limitations. For example, histopathological assessments were limited to the liver and kidneys, and the levels of heavy metals, other herbal contaminants, and pesticides were not ascertained.

## Conclusion

GLES at the highest dose of 1000 mg/kg BW shows signs of toxicity after repeated dose 90-day oral toxicity study. Therefore, further investigations are needed to reach more specific conclusions about the safety of ingesting high doses of GLES for long periods of time.

## Data Availability

The datasets used and/or analysed during the current study are available from the corresponding author on reasonable request.

## Abbreviations

AC: atherogenic coefficient
AIP: atherogenic index of plasma
ALB: albumin
ALP: alkaline phosphatase
ALT: alanine aminotransferase
AST: aspartate aminotransferase
BUN: blood urea nitrogen
BW: body weight
Ca: calcium
CHOL: total cholesterol
CREA: creatinine
CRI-2: Castelli’s Risk Index-2
CRR: cardiac risk ratio
EDTA: ethylene diamine tetra acetic acid
FDA: Food and Drug Administration
GL: *Gongronema latifolium* Benth.
GLES: ethanolic leaf extract of GL
GLO: globulin
HDLC: high density lipoprotein cholesterol
HGB: hemoglobin
LD_50_: median lethal dose
LDLC: low density lipoprotein cholesterol
LYM: lymphocyte absolute value
MCH: mean corpuscular hemoglobin
MCHC: mean corpuscular hemoglobin concentration
MCV: mean corpuscular volume
NEU: neutrophils absolute value
NOAEL: No Observed Adverse Effect Level
OECD: Organisation for Economic Co-Operation and Development
P: phosphorus
PCV: packed cell volume
PLT: platelets
RBC: red blood cells
RDW: red cell distribution width
SD: Sprague Dawley
SEM: standard error of the mean
TG: triglycerides
TP: total protein
USM: Universiti Sains Malaysia
WBC: white blood cells
WHO: World Health Organization
